# Pharmacokinetic Profile of Two Active Dipyrone Metabolites, 4-Methylaminoantipyrine (MAA) and 4-Aminoantipyrine (AA), Following Intravenous Administration in Dogs: A Preliminary Study

**DOI:** 10.3390/ani15111666

**Published:** 2025-06-05

**Authors:** Andressa N. Mouta, Kathryn N. Arcoverde, Naftáli S. Fernandes, Yanna D. B. Passos, Caio V. A. de Oliveira, Robson A. Honorato, Gabriel Araujo-Silva, Valéria V. de Paula

**Affiliations:** 1 Departamento de Ciência Animal, Universidade Federal Rural do Semi-Árido (UFERSA), Mossoró 59625-900, RN, Brazil; andressanmouta@hotmail.com (A.N.M.); kathrynnobrega@gmail.com (K.N.A.); nafta_le@hotmail.com (N.S.F.); yannapassosanest@gmail.com (Y.D.B.P.); caio.almeidavet@gmail.com (C.V.A.d.O.); 2 Centro Universitário INTA–UNINTA, Sobral 62050-100, CE, Brazil; honorato.ra@gmail.com; 3 Escola de Química, Universidade do Estado do Amapá, Macapá 68900-070, AP, Brazil; gabriel.silva@ueap.edu.br

**Keywords:** analgesia, chromatography, metamizole, metabolism

## Abstract

This study aimed to determine the pharmacokinetic profile of the active dipyrone metabolites, 4-methylaminoantipyrine and 4-aminoantipyrine, following intravenous administration in dogs. Eleven mixed-breed dogs received a 25 mg·kg^−1^ dipyrone dose and blood samples were collected at 0, 5, 15, 30 and 45 min, as well as at 1, 1.5, 2, 4, 6, 8, 10, 12, 24, 36 and 48 h. Plasma concentrations of both metabolites were analyzed by ultra-performance liquid chromatography coupled to mass spectrometry. The PKSolver 2.0 and GraphPad Prisma 10 software programs were used for pharmacokinetic and statistical analyses, applying a Principal Component Analysis for MAA and descriptive statistics for both metabolites. Two groups were noteworthy concerning MAA: slow metabolizers (SM) and normal/rapid metabolizers (NM). Significant differences were observed between half-life (T_½_) and MRT_0_inf obs_ values between the MAA groups. The T_½_ and MRT_0_info obs_ were 44.44 ± 11.74 and 32.62 ± 16.53 h for the SM group and 11.25 ± 5.37 and 7.44 ± 4.25 h for the NM group, respectively. The C_max_ of AA was 2.80 ± 1.43 µg mL^−1^. Metabolites were detectable for 48 h in all animals for MAA and seven for AA. These findings suggest that metamizole reaches analgesia plasma concentrations associated with cyclooxygenase inhibition with few adverse effects in dogs. However, additional pharmacogenetic and pharmacotherapeutic monitoring studies are required.

## Simple Summary

Dipyrone is widely used to control pain. However, although the standard dose for routine use is 25 mg·kg^−1^, no studies addressing the pharmacokinetics of this drug employing ultra-performance liquid chromatography coupled to mass spectrometry (UPLC-MS/MS) are available to date. Thus, the aim of this study was to determine the pharmacokinetic profile of the active metabolites of dipyrone 4-methylaminoantipyrine (MAA) and 4-aminoantipyrine (AA) administered intravenously, at 25 mg·kg^−1^, in eleven mixed-breed dogs (weighing 14.43 ± 2.86 kg). Serial blood samples were collected after the drug administration, stored at −80 °C and analyzed by high-performance chromatography coupled to mass spectrometry. A Principal Component Analysis (PCA) indicated two groups of metabolizers, fast and slow, for MAA. This demonstrates metabolism variability within the same group of mixed-breed dogs. Furthermore, the minimum effective concentrations for promoting analgesia in humans were reached for dipyrone metabolites. These results suggest that dipyrone achieves therapeutic plasma concentration with minimal adverse effects. However, further pharmacogenetic and pharmacotherapeutic studies are required to refine dosing recommendations.

## 1. Introduction

Pain control in animals is of utmost importance in clinical practice, promoting physical condition and quality of life improvements, both of which are essential for their well-being [1]. Dogs are constantly subjected to surgical procedures or treated for pathological conditions that culminate in pain, requiring adequate pain control [2]. Thus, the use of analgesic drugs such as nonsteroidal anti-inflammatory drugs (NSAIDs), opioids, and non-opioid analgesics represents an important practice for treating acute pain in small animals [3].

Metamizole, more commonly known as dipyrone, is an analgesic indicated for pain treatment in both humans and animals [4]. Although previously classified as an NSAID due to non-selective inhibition of cyclooxygenase (COX), with emphasis on COX-3 and displaying weak COX-1 and COX-2 inhibition, metamizole exhibits weak anti-inflammatory action and promotes the activation of opioidergic pathways, thus being currently classified as a non-opioid analgesic [5,6]. However, its use is prohibited in countries such as the United States, Japan and Australia due to side effects like agranulocytosis and aplastic anemia in humans, although several countries in Europe, Asia and South America, including Brazil, continue to market this drug [6,7].

Pharmacokinetics, defined as the study of drug absorption, distribution, metabolism, and excretion (ADME), is essential for understanding drug behavior in both physiological and pathological conditions. Studies in this regard are useful for appraising certain parameters, such as dosage regimen and drug behavior following administration [8]. Despite restrictions on the use of dipyrone in many countries, pharmacokinetic studies concerning this drug in different animal species are paramount for evaluating applied doses, administration intervals and plasma concentrations [9,10,11,12,13,14].

Metamizole is a prodrug rapidly hydrolyzed in plasma to its main metabolite, 4-methylaminoantipyrine (MAA) [15]. This metabolite is, in turn, metabolized by cytochrome P450 (CYP) enzymes in the liver into 4-formylaminoantipyrine (FAA) through oxidation processes and into 4-aminoantipyrine (AA) by demethylation [16]. Subsequently, AA can be acetylated to 4-acetylaminoantipyrine (AAA) by N-acetyltransferase 2 (NAT2) [15,17]. The analgesic character of this drug is mainly attributed to its two active metabolites, MAA and AA [18].

Regarding research on dipyrone pharmacokinetics in dogs, Kalchofner Guerrero et al. [19] described the plasma concentrations of active metamizole metabolites in relation to time via oral administration in dogs, but did not describe their complete pharmacokinetic profiles. Only Giorgi et al. [9] reported this, for female Labradors, in which different administration routes of a 25 mg kg^−1^ dose, including by the intravenous route, were evaluated using high-performance liquid chromatography (HPLC) coupled to an ultraviolet detector (HPLC-UV).

In this context, this study aimed to evaluate the pharmacokinetic profiles of two active dipyrone metabolites (MAA and AA) administered intravenously at 25 mg kg⁻^1^ in male and female mixed-breed dogs through ultra-performance liquid chromatography coupled with mass spectrometry (UPLC-MS/MS). In comparison, Giorgi et al. [9] assessed only a single breed, detecting interindividual metabolism changes. Furthermore, the use of liquid chromatography coupled with mass spectrometry (LC-MS) offers significant advantages over the HPLC-UV method, including greater metabolite detection and quantification sensitivity and specificity, allowing for more accurate and detailed pharmacokinetic profile assessments.

## 2. Materials and Methods

### 2.1. Animals and Study Preparation

This study was approved by the Federal Rural Semi-Arid University Animal Use Ethics Committee (CEUA/UFERSA), under number 07/2023 (2 May 2023). Eleven adult dogs (five females and six males), with a minimum age of 1 year and a maximum of 5 years (1.72 ± 1.35), intact and healthy, weighing 14.43 ± 2.86 kg, all mixed breeds, were obtained from a non-governmental organization (NGO), located in Caraúbas, Rio Grande do Norte, Brazil. The consent and authorization form to carry out the study was signed by the people responsible for the animals.

The dogs were grouped in kennels on the premises of UFERSA’s Internal Veterinary Medicine Laboratory and subjected to 8-h food and 4-h water fasts. All animals underwent physical and laboratory examinations (complete blood counts and serum biochemistry-urea, creatinine, total protein, albumin, alanine aminotransferase and alkaline phosphatase) and were included in the study after being certified as healthy.

The 11 animals were manually restrained, and the cephalic vein region underwent trichotomy and antisepsis for insertion of a 20 G catheter, coupled to a 3-way stopcock to maintain viable venous access. All animals were subjected to the same dipyrone treatment (D-500^®^, Zoetis, São Paulo, Brazil) administered intravenously at 25 mg·kg^−1^ for 2 min using an infusion pump (DigiPUMP SR 31X-Digicare^®^, Rio de Janeiro, Brazil).

All animals were evaluated for any sign of behavioral changes and adverse effects, such as salivation, sweating, muscle spasms, restlessness and tachypnea, assessing symptom onset and duration.

### 2.2. Pharmacokinetic Study

To determine plasma MAA and AA concentrations, 4 mL of blood was collected from the cephalic vein of all animals, at the following time points: 0 (prior to drug administration), 5, 15, 30, 45 min and 1, 1.5, 2, 4, 6, 8, 10, 12, 24, 36 and 48 h. The samples were stored in tubes containing ethylenediaminetetraacetic acid (EDTA) and centrifuged at 3500 rpm for 10 min to obtain blood plasma, which was then stored in cryogenic tubes at −80 °C for later analysis by UPLC-MS/MS.

### 2.3. Sample Extraction Procedures

Aliquots of the obtained plasma samples (250 µL) were mixed with 10 µL ofa 0.1 mg mL^−1^ metoprolol solution (internal standard) and 800 µL of acetonitrile, followed by vortexing for 60 s and centrifugation for 10 min at 10,000 rpm. The supernatants (900 µL) were transferred to vials, and 5 µL were injected into the UPLC-MS/MS chromatographic system, consisting of a Nexera 2 UPLC coupled to an LCMS-8040 mass spectrometry detector (Shimadzu, Kyoto, Japan) and a Phenomenex UPLC Luna Omega C18 column (1.6 μm, 2.1 × 50 mm) (Phenomenex, Torrance, CA, USA).

### 2.4. Analytical UPLC-MS/MS Conditions

The mobile phase comprised acetonitrile and a 0.1% formic acid solution (75:25, *v*/*v*) at 0.3 mL min^−1^. The run time was set at 2.0 min, and the injected sample volume was 5.0 μL. The column temperature was adjusted to 40 °C, and the autosampler refrigerator was set to 5 °C.

The mass spectrometer was set to multiple reaction monitoring (MRM) mode in positive ionization mode (ESI+). The applied collision energy and cone voltage were 28 and 25 V, respectively. The nebulizer gas flow rate was 3 L min^−1^, using argon as the collision gas at a flow rate of 15 L min^−1^. The mass spectrometer was set to monitor the band transition of the parent and daughter ions. A dwell time of 0.1 s was established, and MRM data were acquired and analyzed using the Labsolution software version 5.114 (Shimadzu, Kyoto, Japan).

The mass and charge ratio (*m*/*z*) of the main analyzed ions were MAA (218.1), AA (204.1) and metoprolol (268.2), and for the daughter ions, MAA (159.1), AA (77.00) and metoprolol (131.0).

### 2.5. Validation

The analytical method was revalidated according to the International Conference on Harmonization ICH (2018) and the National Health Surveillance Agency [20] criteria. A drug-free plasma was spiked with a standard solution to prepare a calibration curve. Quality control samples (spots) were prepared at low (80 ng mL^−1^), medium (5600 ng mL^−1^) and high (40,000 ng mL^−1^) levels for both MAA and AA and used to determine absolute recoveries and intra- and inter-day precisions. Selectivity was assessed by preparing a curve using the lower limit of quantification (LLOQ), established as 0.076 µg mL^−1^ for both metabolites in drug-free plasma. Stability concerning the biological matrix at −70 °C, bench temperature at room temperature (20 °C) and samples were assessed using an autosampler.

### 2.6. Pharmacokinetic Analysis

Pharmacokinetic parameters were calculated by applying a non-compartmental model with PKSolver 2.0 software. The observed variables were as follows: maximum plasma concentration (C_max_), plasma concentration at time zero (C_0_), time to reach C_max_ (T_max_), half-life (T_½_), area under the plasma concentration curve from time zero to the time of the last measurable concentration (AUC_0→t_) and the extrapolation of AUC to infinity (AUC_0→∞_), the ratio between AUC_0→t_ and AUC_0→∞_, the area under the curve from the first time of zero to the last measurable concentration AUMC_0→t_; the mean residual time from moment zero to infinity (MRT_0→ inf_), volume of distribution (Vd), which is the estimated volume of distribution based on the AUC, the volume of distribution at steady state (Vss), the clearance (Cl), the terminal phase rate constant (λz).

### 2.7. Statistical Analyses

Descriptive statistical analyses were performed using Python (version 3.10). Data were initially tested for normality using the Kolmogorov–Smirnov test. Comparative statistics were performed applying an Analysis of Variance (ANOVA) with a post hoc Tukey test and Mann–Whitney between groups for MAA data (SL, NM and all dogs), with significance set at *p* < 0.05. A post hoc *t*-test for independent samples was performed to calculate Cohen’s d and statistical power, assessing the magnitude and reliability of differences between metabolizer groups (SL and MN groups). In addition, a Principal Component Analysis (PCA) was performed for MAA pharmacokinetic variables. Data were presented as means ± standard deviations for AA and MAA, and the median (maximum–minimum) was also presented for MAA.

## 3. Results

The method was revalidated to ensure data accuracy according to other studies [14,21,22]. All calibration curves presented R^2^ greater than 0.99, demonstrating linearity between equipment responses and curve concentrations. The method also presented good selectivity, reproducibility and repeatability, with relative standard deviations below 5%. The samples were stable under the analysis conditions.

**Figure 1:**
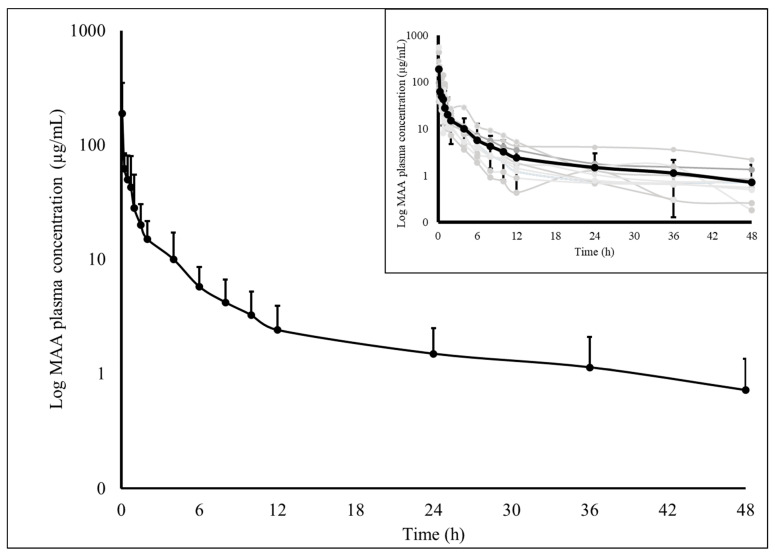
Log of the plasma concentration of 4-methylaminoantipyrine (MAA) as a function of time, at 25 mg kg^−1^ metamizole administered intravenously in dogs. The individual pharmacokinetic profiles of all animals are highlighted (grey lines). The MAA results are expressed as means and standard deviations (black lines) (n = 11).

**Figure 2:**
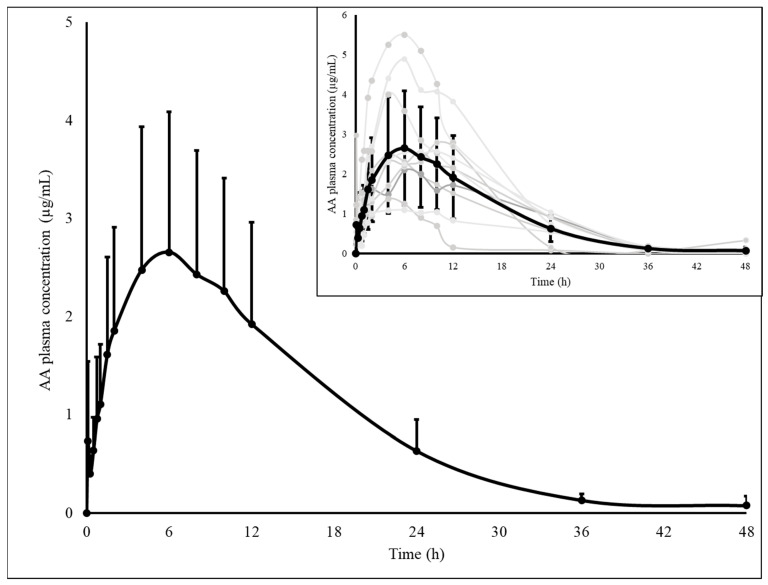
Plasma concentration of 4-aminoantipyrine (AA) as a function of time, at 25 mg kg^−1^ metamizole administered intravenously. The individual pharmacokinetic profiles of all animals are highlighted (grey lines). The AA results are expressed as means and standard deviations (black lines) (n = 11).

After intravenous metamizole administration, both MAA and AA metabolites were analyzed for 48 h in all dogs, with MAA quantified at this time in all animals, while AA was quantified in seven. The plasma concentrations of both metabolites over time are described in Figure 1 and Figure 2.

Regarding general pharmacokinetic parameters, the data are expressed as the means ± standard deviations (Table 1) and compared to the results reported by Giorgi et al. [9] for dogs. All animals showed optimal correlations in the non-compartmental pharmacokinetic model employing the applied dose (R^2^ > 0.7).

**Figure 3:**
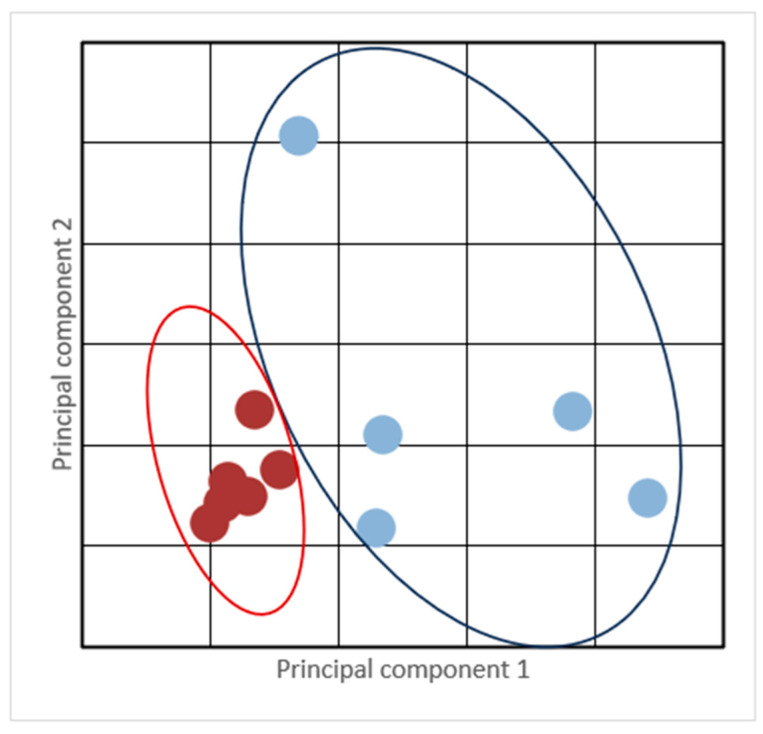
Principal Component Analysis (PCA) was performed for MAA, assessing the investigated pharmacokinetic parameters (C_max_, T_½_, MRT _0-inf_obs_, AUC _0-t_, Vd, Cl). Normal/fast metabolism pattern (NM group; red) and slow metabolism (SM group; blue).

A Principal Component Analysis (PCA) was performed (Figure 3), demonstrating the correlation between the different pharmacokinetic parameters in relation to MAA (Cmax, T_½_, MRT _0-inf_obs_, AUC _0-t_, Vd, Cl). This analysis indicated two groups of animals: the NM group (n = 6) represented in red (normal/fast metabolizers), and the SM group (n = 5) represented in blue (slow metabolizers).

Table 2 depicts MAA metabolism differences among the analyzed animals. Five animals (two males and three females) presented a slow metabolism pattern (SM group), while six (three males and three females) presented a normal/fast metabolism pattern (NM group), demonstrated mainly by significant differences in T_½_, and MRT0-inf_obs values. The data are expressed as means ± standard deviations and medians (maximum–minimum). Effect size and power analyses confirmed the robustness of statistically significant differences for T½, MRT_0_–inf_obs, and AUC_0_–t/AUC_0_–inf_obs between metabolizer groups. These parameters showed very large effect sizes (Cohen’s d > 2.0) and high statistical power (>88%).

Regarding behavioral and adverse effects, three animals presented tachypnea, two belonging to the SM group and one to the NM group, at the beginning of the drug administration, with an average duration of 15 min.

## 4. Discussion

This is a first-time report concerning the evaluation of active dipyrone metabolite pharmacokinetics in male and female dogs employing the UPLC-MS method. Although one study describing pharmacokinetic parameters by different routes, including intravenous administration in dogs, has been carried out by Giorgi et al. [9], important differences in their sampling design and applied analytical method compared to the present study are noteworthy. A 25 mg kg^−1^ dose was chosen due to its routine use in dogs in clinical practice. One study carried out in the postoperative period of ovariohysterectomy (OH) surgery in bitches assessed the analgesic effects and physiological and hematological parameters of 15, 25 and 35 mg kg^−1^ dipyrone doses administered intravenously [23]. The 25 and 35 mg kg^−1^ doses were efficient in promoting analgesia with no hematological and clinical parameter changes, while the 15 mg kg^−1^ dose did not promote satisfactory postoperative analgesia. The authors of that study also emphasized that, since no pain score increases were observed up to 8 h after administration, it is likely that metamizole administered in this range may be able to provide adequate analgesia.

In another clinical study, the administration of dipyrone alone at 25 mg kg^−1^ led to a higher degree of analgesia in bitches undergoing the same OH procedure, as well as a reduced need for rescue analgesics during the postoperative period [24]. These findings reinforce the choice of the 25 mg kg^−1^ dose in the present study and suggest that dipyrone exhibits adequate clinical efficacy when applied in animals after painful stimulation, although pharmacokinetic parameter investigations are necessary in this sense.

Table 1 presents significant differences in the pharmacokinetic parameters of the metabolites MAA and AA when compared to the study by Giorgi et al. (2018) [9], which can largely be attributed to the greater interindividual variability resulting from the inclusion of both sex and multiple dog breeds in the present study, as opposed to the use of a single breed and only female in the previous research [9,24]. This diversity led to greater data dispersion, as evidenced by the wide variation in MAA half-life (T½), which reached 26.39 ± 19.29 h compared to 5.94 ± 2.54 h reported by Giorgi, as well as significantly higher values of C_max_ (203.68 ± 159.24 µg/mL vs. 21.80 ± 2.45 µg/mL) and AUC_0₋t_ (205.71 ± 108.18 µg/mL·h vs. 45.34 ± 9.64 µg/mL·h). For AA, although the T½ values were similar (6.72 ± 1.66 h vs. 8.05 ± 2.56 h), the C_max_ was more than twice as high (2.80 ± 1.43 µg/mL vs. 1.29 ± 0.21 µg/mL), and the AUC_0₋t_ was also considerably greater (43.87 ± 18.94 µg/mL·h vs. 17.97 ± 2.91 µg/mL·h). The lower clearance observed for MAA (147.67 ± 103.85 mL/h/kg vs. 552.43 ± 98.34 mL/h/kg) and the increased apparent clearance for AA (712.76 ± 45.23 mL/h/kg vs. 1224.03 ± 228.74 mL/h/kg) further reflect this inter-animal heterogeneity. These findings highlight the relevance of inter-individual, particularly genetic and physiological, variability in pharmacokinetic studies and reinforce the importance of including different breeds in more representative preclinical assessments.

The minimum MAA concentrations to promote 50% cyclooxygenase enzyme inhibition (IC50–COX-1 and COX-2) have been described in humans, comprising an important tool used to determine drug potency, with values of 0.553 µg mL^−1^ for IC50-COX1 and 0.926 µg mL^−1^ for IC50-COX2 [25,26]. This indicates that achieved plasma concentrations promoted minimal COX-1 and COX-2 inhibition in both Giorgi et al. [9] and in the present study, suggesting that the main active dipyrone metabolite presents high inhibitory effects against these enzymes. COX-1 and COX-2 are two isoenzymes, the former considered constitutive and physiological, while the latter is inducible [27]. These evaluations comprise assessments on analgesic dipyrone mechanisms of action, as this agent presents a complex inhibition mechanism regarding COX isoenzymes (1, 2 and 3), although it also acts quickly on opioid receptors and on the cannabinoid system [5,28].

A study comparing a 25 mg kg^−1^ dipyrone dose every 24 h and 12.5 mg kg^−1^ every 12 h in cats undergoing OH demonstrated COX-1 and COX-2 inhibition at both doses, with equally effective analgesia promotion [29]. These data reinforce the need to evaluate the applied dose regimen, supporting metamizole metabolism differences between dogs and cats.

Regarding pharmacokinetic parameters, the T_½_ value observed for MAA in this study was considerably higher (26.39 h) (Table 1) than those previously reported for dogs (5.94 h) [9], cats (4.42 h) [10], and donkeys (3.62 h) [14] at the same dose by the same administration route. These findings may be associated with differences between species and individuals, as well as in enzymatic expression, and analytical method sensitivity, since mass spectrometry, a very sophisticated method with high selectivity and sensitivity for analyte analysis and separation [30], was employed herein. A direct comparison with the findings reported by Giorgi et al. [9] for dogs highlights sex, breed and size differences; in the aforementioned study, female Labradors averaging 36 to 42 kg were analyzed, in contrast to the present study, in which both male and female dogs of mixed breeds averaging 14.43 kg were used.

Human studies suggest differences in the activity of some CYP enzymes, such as CYP3A4, which is responsible for over 50% of the metabolism of commonly used drugs (including metamizole), between genders. In some studies, greater CYP3A4 activity has been detected in women compared to men, although some in vitro data are used, while in other studies, no significant differences related to CYP3A4 activity at the gastrointestinal level were suggested between men and women [31]. Studies on dog pharmacogenetics report pharmacokinetics and pharmacodynamics differences between breeds, so the same treatment used in different breeds may result in different findings [32].

Some animals presented slow metabolism (SM) behavior, which increased T_½_ (44.44 h), MRT (32.62 h), C_max_ (238.29 µg mL^−1^) and Vd (6.32 mL kg^−1^) values compared to normal/rapid metabolizers (NM), with values of 11.25 h, 7.44 h, 174.84 µg mL^−1^ and 2.77 mL kg^−1^, respectively. In humans, CYP3A4, CYP2B6, CYP2C8 and CYP2C9 are mainly responsible for MAA N-demethylation, transforming this compound into AA [15,16], while oxidation of MAA to FAA is carried out by nonspecific CYP enzymes.

**Figure 4:**
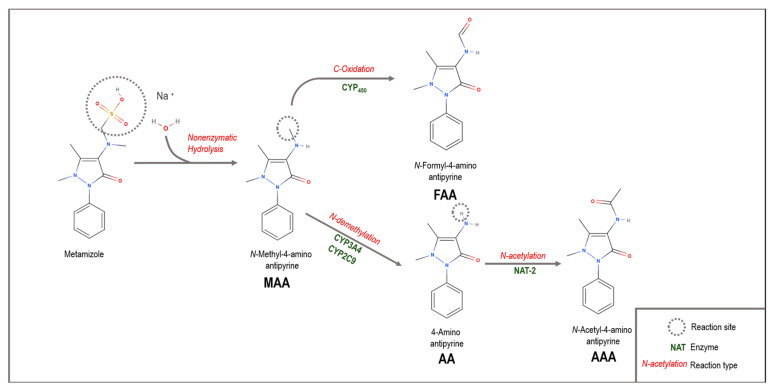
Metamizole metabolism.

In dogs, this may justify the metabolism differences observed herein, as mixed-breed animals do not present a specific pattern, resulting in potential differences in the expression of different CYP genes, leading to fast or slow metabolization profiles. These data were also suggested by Fux et al. [33] when applying 40 mg kg^−1^ metamizole in eight calves 10 min prior to general anesthesia for umbilical surgery, reporting that five animals showed faster MAA to AA metabolization. The authors discuss individual differences in metabolic hepatic activity and CYPs, which present interindividual expression and activity variabilities in cattle [34]. However, it is important to note that Fux et al. [33] analyzed animals who had also been administered other drugs, such as meloxicam, ketamine and xylazine, which can lead to metabolism variations. Therefore, these differences between CYP activities may justify the metabolism differences observed herein between individuals, as metamizole metabolism is dependent on these enzymes through N-demethylation and C-oxidation (MAA to FAA) (Figure 4).

The detected plasma AA concentrations in this study were lower than MAA concentrations, similar to findings reported for cats [10,35], dogs [9] and horses [12] administered the same dose, and for donkeys administered different doses [14]. This suggests that the MAA to AA metabolism occurs similarly in different animal species, i.e., the production of AA and the conversion of AA into AAA do not present variable metabolism profiles between species or individuals due to low AA concentrations. The AUC was also lower, as AA is a product of MAA metabolism [15].

The plasma concentration of MAA was rapidly reached due to the abrupt entry of metamizole into the vascular compartment, which is quickly hydrolyzed into MAA. The MAA C_0_ value was considerably higher in this study (456.93 µg mL^−1^) compared to the C_max_ value reported by Giorgi et al. [9]. These differences reflect the employed analytical method and the physical and genetic characteristics of the animals assessed herein. The MAA AUC was quite high, above other studies, representing high drug exposure over time in the present study.

Regarding pharmacokinetic AA data, the T_½_ value reported in the present study was of 6.72 h, comparable to dogs (8.05 h) as reported by Giorgi et al. [9], as well as donkeys (7.11 h) [14] and cats (13.66 h) as reported by de Paula et al. [10], reinforcing a slower metamizole metabolism in felines. The analyzed AA parameters differed only slightly from data previously reported for dogs, demonstrating similar AA metabolism profiles in both studies carried out on dogs and suggesting that canine metabolic routes do not vary significantly. MAA and AA pharmacokinetic and metabolism profiles are quite distinct, with AA following a model similar to the intramuscular administration profile, unlike MAA, which follows an intravenous administration profile. This is due to the transformation of MAA into AA. In this sense, Arcoverde et al. [21] carried out pharmacotherapeutic monitoring of dipyrone in donkeys, reporting AA accumulation during dipyrone administration carried out every 12 h. These data should be assessed in dogs to verify whether the same is observed in canines and evaluate the clinical implications.

Regarding adverse effects, episodes of vomiting and salivation were not observed in dogs, although they have been reported in cats [10,35], with the only effect observed herein encompassing increased respiratory rates, lasting an average of 15 min in three animals, two from the SM group and one from the NM group. It is important to highlight that these animals presented agitated behavior, which may have contributed to the occurrence of this effect and is not clinically relevant. No donkeys [14] or horses [12] presented adverse effects. The clinical studies carried out by Imagawa et al. [23] in dogs and Pereira et al. [29] in cats did not report relevant adverse effects regarding hematological changes, while Zanuzzo et al. [24] detected transient platelet aggregation inhibition. This is noteworthy because dipyrone is banned in several countries due to the hematological effects found in humans [16]. Thus, further studies correlating metamizole pharmacokinetic profiles with physiological and hematological changes should be carried out, as well as more studies employing different doses and administration intervals to determine alternatives for different metamizole doses in dogs.

## 5. Conclusions

The findings reported herein suggest that achieved plasma concentrations may promote analgesic and anti-inflammatory effects associated with cyclooxygenase inhibition, with few adverse effects in dogs. Two active dipyrone metabolites were formed in dogs, and the analytical UPLC-MS method was deemed adequate for their detection. Further studies involving pharmacokinetics, pharmacogenetics (concerning the polymorphism associated with dipyrone metabolism) and pharmacotherapeutic monitoring are necessary to determine T½ differences in order to define an appropriate dipyrone dose range for dogs.

## Figures and Tables

**Table 1:** Pharmacokinetic parameters of 4-Methylaminoantipyrine (MAA) and 4-Aminoantipyrine (AA) following intravenous metamizole administration at 25 mg kg^−1^ in dogs (n = 11) compared to the study carried out by Giorgi et al. [9].

		MAA	MAA (Giorgi et al., 2018) [9]	AA	AA (Giorgi et al., 2018) [9]
Parameter	Unit	Means ± SD	Means ± SD	Means ± SD	Means ± SD
**Lambda_z**	1/h	0.06 ± 0.08	0.13 ± 0.04	0.11 ± 0.03	0.09 ± 0.03
**T _½_**	h	26.39 ± 19.29	5.94 ± 2.54	6.72 ± 1.66	8.05 ± 2.56
**C_max_**	µg/mL	203.68 ± 159.24	21.80 ± 2.45	2.80 ± 1.43	1.29 ± 0.21
**C_0_**	µg/mL	409.23 ± 524.72	-	-	-
**T_max_**	h	-	-	6.18 ± 2.27	5.33 ± 1.63
**AUC _0-t_**	µg/mL × h	205.71 ± 108.18	45.34 ± 9.64	43.87 ± 18.94	17.97 ± 2.91
**AUC _0-inf_obs_**	µg/mL × h	240.02 ± 127.38	46.79 ± 9.15	-	21.08 ± 4.34
**AUC _0-t/0-inf_obs_**		0.88 ± 0.12	-	-	-
**AUMC _0-inf_obs_**	µg/mL × h^2^	5164.94 ± 5735.48	246.57 ± 31.01	540.32 ± 245.01	273.97 ± 117.74
**MRT 0-inf_obs**	h	18.88 ± 17.07	5.37 ± 0.81	12.09 ± 2.79	12.62 ± 3.13
**Vd**	(L/kg)	4.38 ± 3.62	4.95 ± 0.99	-	-
**Vss**	(L/kg)	-	-	6.72 ± 3.74	13.85 ± 3.68
**Cl**	(mL/h/kg)	147.67 ± 103.85	552.43 ± 98.34	712.76 ± 45.23	1.224.03 ± 228.74

Legend: Lambda_z: terminal phase rate constant; T_1/2_: half-life; C_max_: maximum plasma concentration; C_0_: plasma concentration at time zero; T_max_: time to reach maximum plasma concentration; C_max_: maximum plasma concentration; AUC_0→t_: area under the curve from 0 to the last measurement; AUC_0→∞_: area under the curve from 0 to infinity; AUC_0→t_/AUC_0→∞_: the ratio between AUC_0→t_ and AUC_0→∞_; AUMC_0→t_: area under the curve from the first moment from zero to the last measurable concentration; MRT_0→∞_: mean residual time from 0 to infinity; Vd: apparent volume of distribution; Vss: volume of distribution at steady state; Cl: total body clearance.

**Table 2:** Pharmacokinetic parameters determined for 4-Methylaminoantipyrine (MAA) following intravenous metamizole administration at 25 mg kg^−1^ in dogs (n = 11), all of mixed breeds, with metabolism differences detected among individuals, categorized as slow metabolizers (SL) (n = 5, two males and three females) and normal/rapid metabolizers (NM) (n = 6, three males and three females).

MAA		All Dogs (n = 11)	Slow Metabolized (SL) (n = 5)		Normal/Quick Metabolized (NM) (n = 6)	
Parameter	Unit	Means ± SD	Means ± SD	Median (Max–Min)	Means ± SD	Median (Max–Min)
**Lambda_z**	1/h	0.06 ± 0.08	0.02 ± 0.004	0.017 (0.020–0.011)	0.09 ± 0.09	0.05 (0.29–0.04)
**T _½_**	h	26.39 ± 19.29	44.44 ± 11.74 *	38 (58.35–33.57) *	11.25 ± 5.37	12.58 (16.8–2.35)
**C_max_**	µg/mL	203.68 ± 159.24	238.29 ± 197.92	223.68 (548.74–44.13)	174.84 ± 131.07	149.14 (428.98–71.78)
**C_0_**	µg/mL	409.23 ± 524.72	606.69 ± 757.10	434.48 (1912.98–38.00)	244.67 ± 143.60	211.38 (493.71–77.45)
**T_max_**	h	-	-		-	-
**AUC _0-t_**	µg/mL×h	205.71 ± 108.18	239.78 ± 88.95	250.42 (336.07–103.95)	177.32 ± 122.27	166.84 (404.65–67.37)
**AUC _0-inf_obs_**	µg/mL×h	240.02 ± 127.38	306.76 ± 106.84 *	326.29 (441.35–148.5) *	184.41 ± 123.09	174.99 (407.78–69.99)
**AUC _0-t/0-inf_obs_**		0.88 ± 0.12	0.78 ± 0.1 *	0.76 (0.90–0.65) *	0.95 ± 0.04	0.95 (0.99–0.90)
**AUMC _0-inf_obs_**	µg/mL×h^2^	5164.94 ± 5735.48	9733.64 ± 5762.53	6899.49 (17,240.51–3583.31)	1357.68 ± 979.22	1281.33 (2857.61–153.47
**MRT _0-inf_obs_**	h	18.88 ± 17.07	32.62 ± 16.53 *	32.43 (52.83–12.97) *	7.44 ± 4.25	7.18 (14.09–2.02)
**Vd**	(L/kg)	4.38 ± 3.62	6.32 ± 4.29	4.71 (13.62–2.74)	2.77 ± 2.13	2.33 (6.77–1.06)
**Cl**	(mg/kg)/(ug/mL)/h	0.15 ± 0.10	0.09 ± 0.04	0.07 (0.16–0.05)	0.19 ± 0.12	0.14 (0.35–0.06)

Data are presented as means ± standard deviations and medians (maximum–minimum). Legend: Lambda_z: terminal phase rate constant; T_½_: half-life; C_max_: maximum plasma concentration; C_0_: plasma concentration at time zero; T_max_: time to reach maximum plasma concentration; C_max_: maximum plasma concentration; AUC_0→t_: area under the curve from 0 to the last measurement; AUC_0→∞_: area under the curve from 0 to infinity; AUC_0→t_/AUC_0→∞_: the ratio between AUC_0→t_ and AUC_0→∞_; AUMC_0→t_: area under the curve from the first moment from zero to the last measurable concentration; MRT_0→∞_: mean residual time from 0 to infinity; Vd: apparent volume of distribution; Vss: volume of distribution at steady state; Cl: total body clearance. * Indicates statistical difference between the SL group and the NM group/all dogs (*p* < 0.05).

## Data Availability

No new data were created or analyzed in this study. Data sharing is not applicable to this article.
